# Risk factors and prognosis of malignant peritoneal mesothelioma with paraneoplastic syndrome

**DOI:** 10.1186/s12957-024-03312-w

**Published:** 2024-01-24

**Authors:** Xin-Li Liang, Yan-Dong Su, Xin-Bao Li, Yu-Bin Fu, Ru Ma, Rui Yang, He-Liang Wu, Yan Li

**Affiliations:** 1 Department of Peritoneal Cancer Surgery, Beijing Shijitan Hospital, Capital Medical University, Haidian District, No. 10 Tieyi Road, Yangfangdian Street, Beijing, 100038 China; 2 Department of Peritoneal Cancer Surgery, Beijing Shijitan Hospital, Peking University Ninth School of Clinical Medicine, Beijing, 100038 China; 3 Department of Surgical Oncology, Beijing Tsinghua Changgung Hospital, Tsinghua University, Beijing, 102218 China

**Keywords:** Malignant peritoneal mesothelioma, Paraneoplastic syndrome, Thrombocytosis, Neoplastic fever, Prognosis

## Abstract

**Background:**

Malignant peritoneal mesothelioma (MPM) is a rare and highly aggressive tumor. Its clinical manifestations are diverse, and the symptoms are not specific. Some patients will develop paraneoplastic syndrome (PS) during the disease course. This study aims to analyze the risk factors of PS in patients with MPM and their impacts on prognosis.

**Methods:**

The clinical data of MPM patients who underwent cytoreductive surgery plus hyperthermic intraperitoneal chemotherapy (CRS + HIPEC) at our center from June 2015 to May 2023 were retrospectively analyzed. MPM patients were divided into PS group and non-PS group according to the diagnostic criteria. Univariate and multivariate analyses were performed to explore the risk factors of PS in MPM patients, and to analyze the impact of PS on prognosis.

**Results:**

There were 146 MPM patients in this study, including 60 patients (41.1%) with PS and 86 patients (58.9%) without PS. The highest incidence of PS was thrombocytosis (33.6%), followed by neoplastic fever (9.6%). Univariate analysis revealed 8 factors (*P* < 0.05) with statistically significant differences between the two groups: prior surgical scores, targeted therapy history, Karnofsky performance status score, preoperative carbohydrate antigen (CA) 125 level, vascular tumor embolus, peritoneal cancer index, completeness of cytoreduction (CC) score and intraoperative ascites. Multivariate analysis identified 3 independent factors associated with PS: preoperative CA 125 level, vascular tumor embolus, and CC score. Survival analysis demonstrated that MPM patients with PS had worse prognosis, although PS was not an independent prognostic factor.

**Conclusions:**

PS is not rare in patients with MPM, and is independently associated with preoperative CA 125 level, vascular tumor embolus and CC score. PS often indicates advanced disease and poor prognosis.

## Introduction

Malignant peritoneal mesothelioma (MPM) is a rare and highly aggressive tumor originating from peritoneal mesothelial cells, accounting for 7%-30% of all mesothelioma [1]. In the past, MPM was mainly treated with conservative treatment such as chemotherapy and palliative surgery, but the prognosis was poor, with a median overall survival (OS) of less than 1 year [2]. With the development of cytoreductive surgery (CRS) plus hyperthermic intraperitoneal chemotherapy (HIPEC), the median OS of MPM patients have significantly prolonged to 34 to 92 months [3]. And now CRS + HIPEC has become the standard treatment in epithelioid MPM.

The clinical symptoms of MPM are nonspecific and usually include abdominal pain, bloating, weight loss, and abdominal mass, and a few may manifest as intestinal obstruction and microcirculatory hypercoagulable state [4]. In addition, some patients may also develop a variety of paraneoplastic syndrome (PS), which has various manifestations and present particular difficulties in clinical practice, resulting in missed diagnosis and misdiagnosis [5].

PS is a rare disorder that usually has a complex clinical presentation and is not caused by direct tumor invasion or compression. It arises from tumor secretions of hormones, peptides or cytokines or from immune cross-reactivity between malignant and healthy tissue. PS can involve various systems such as endocrine, nerve, skin, rheumatism, and blood. Furthermore, PS can be present before tumors, so timely diagnosis can help improve the prognosis of malignant diseases [6].

PS associated with MPM is rare, and only a few cases were reported. So, the aim of this study is to retrospectively analyze the clinical data of 146 patients with MPM, summarize the occurrence of PS related to MPM, explore the risk factors of PS, and analyze their impacts on prognosis.

## Patients and methods

### Patients

The study was approved by the Medical Ethics Committee of Beijing Shijitan Hospital affiliated to Capital Medical University (2015-[28]), and all patients signed an informed consent form before treatment. From our prospectively established database on patients with peritoneal malignancy, we selected 146 MPM patients with complete clinical data who underwent CRS + HIPEC from June 2015 to May 2023. All enrolled patients met the inclusion and exclusion criteria of CRS + HIPEC [7]. According to the PS diagnostic criteria, MPM patients were divided into PS group and non-PS group.

### Diagnostic criteria for PS

The diagnosis of PS is difficult to define. It is usually determined by the exclusion method, which must exclude direct invasion or metastasis of tumors, and exclude infection, nutrition, metabolism, anti-tumor treatment and other abnormalities. The specific diagnostic criteria of PS in this study refer to multiple literatures [8–11].

### Study indicators

Major parameters in this study included the following three aspects: (1) Clinicopathological characteristics: gender, age, body mass index (BMI), previous treatment history, prior surgical score (PSS), Karnofsky performance status score (KPS), PS, preoperative carbohydrate antigen (CA) 125 level, pathological type, vascular tumor embolus, lymphatic metastasis, and Ki-67 index. (2) CRS + HIPEC related parameters: peritoneal cancer index (PCI) score, completeness of cytoreduction (CC) score, red blood cell (RBC) transfusion, number of resected organs, and number of resected peritoneal areas. (3) Survival data: survival status and OS.

### Follow‑up

Follow-up was conducted by outpatient visit or telephone interview, covering the following information: survival status, time and cause of death. The last follow-up was June 3, 2023, and the follow-up rate was 100%.

OS was defined as the interval between the date of CRS + HIPEC surgery at our hospital and the end of follow-up or the date of disease-related death.

### Statistical analysis

BM SPSS Statistics for Windows, version 25.0 (IBM Corp., Armonk, NY, USA) was used for data analysis. Measurement data were presented as mean ± standard deviation (SD), and t test was used when the data were in accordance with normal distribution and homogeneity of variance. Enumeration data were presented as frequencies and analyzed using the χ2 and Fisher’s exact tests. Univariate and Logistic regression analysis were used to analyze the risk factors of PS in MPM. Univariate and Cox regression analysis were used to analyze the effect of PS on the prognosis of MPM patients, with *P* < 0.05 considered as statistically significant.

## Results

### Incidence rate of MPM with PS in this study

A total of 146 MPM patients were enrolled in this study, including 60 patients (41.1%) in the PS group and 86 patients (58.9%) in the non-PS group. Among the 60 MPM patients with PS, there were 7 clinical syndrome types, including thrombocytosis (33.6%), neoplastic fever (9.6%), unexplained pain (4.8%), malignant tumor-associated thrombosis (1.4%), anemia (1.4%), hypoglycemia (0.7%), and nephrotic syndrome (0.7%) (Table 1).

**Table 1:** The distribution and incidence of PS in this study

Paraneoplastic syndrome	n (%)^a^
Hematologic system	
Thrombocytosis	49 (33.6)
Malignant tumor-associated thrombosis	2 (1.4)
Anemia	2 (1.4)
Endocrine system	
Hypoglycemia	1 (0.7)
Urinary system	
Nephrotic syndrome	1 (0.7)
Other	
Neoplastic fever	14 (9.6)
Unexplained pain	7 (4.8)

*PS* paraneoplastic syndrome

^a^One patient may have multiple PS

### Major clinicopathological characteristics

The main clinicopathological characteristics were compared between the two groups. In PS *vs.* non-PS groups, PSS 2–3 was 11.7% *vs.* 26.7% (*P* = 0.027); targeted therapy history was 25.0% *vs.* 40.7% (*P* = 0.049); KPS ≥ 80 was 81.7% *vs.* 93.0% (*P* = 0.035); vascular tumor embolus was 35.0% *vs.* 15.1% (*P* = 0.005); increased preoperative CA 125 was 81.7% *vs.* 59.3% (*P* = 0.004). There were no significant differences in other main clinicopathological characteristics between the two groups (Table 2).

**Table 2:** Major clinicopathological characteristics of MPM patients between PS and non-PS groups

Variables	Non-PS group (*n* = 86)	PS group (*n* = 60)	*P* value
Gender, n (%)			0.875
Female	47 (54.7)	32 (53.3)	
Male	39 (45.3)	28 (46.7)	
Age (years), n (%)			0.873
< 60	57 (66.3)	39 (65.0)	
≥ 60	29 (33.7)	21 (35.0)	
BMI (kg/m^2^), n (%)			0.156
< 18.5	5 (5.8)	8 (13.3)	
18.5–23.9	45 (52.3)	34 (56.7)	
≥ 24.0	36 (41.9)	18 (30.0)	
History of surgery, n (%)			0.198
No	31 (36.0)	28 (46.7)	
Yes	55 (64.0)	32 (53.3)	
Previous surgical score, n (%)			**0.027**
0/1	63 (73.3)	53 (88.3)	
2/3	23 (26.7)	7 (11.7)	
History of chemotherapy, n (%)			0.321
No	43 (50.0)	25 (41.7)	
Yes	43 (50.0)	35 (58.3)	
History of radiotherapy, n (%)			0.133
No	86 (100.0)	57 (95.0)	
Yes	0 (0.0)	3 (5.0)	
History of targeted therapy, n (%)			**0.049**
No	51 (59.3)	45 (75.0)	
Yes	35 (40.7)	15 (25.0)	
KPS, n (%)			**0.035**
< 80	6 (7.0)	11 (18.3)	
≥ 80	80 (93.0)	49 (81.7)	
Pathological type, n (%)			0.880
Epithelioid type	65 (75.6)	46 (76.7)	
Non-epithelioid type	21 (24.4)	14 (23.3)	
Vascular tumor emboli, n (%)			**0.005**
No	73 (84.9)	39 (65.0)	
Yes	13 (15.1)	21 (35.0)	
Lymphatic metastasis, n (%)			0.819
No	77 (89.5)	53 (88.3)	
Yes	9 (10.5)	7 (11.7)	
Ki-67 index, n (%)			0.835
≤ 9%	14 (16.3)	9 (15.0)	
> 9%	72 (83.7)	51 (85.0)	
Preoperative CA 125 level (U/mL), n (%)			**0.004**
Normal	35 (40.7)	11 (18.3)	
Increased	51 (59.3)	49 (81.7)	

*MPM* malignant peritoneal mesothelioma, *PS* paraneoplastic syndrome, *BMI* body mass index, *KPS* Karnofsky performance status score

### CRS + HIPEC related parameters

The CRS + HIPEC related parameters were compared between the two groups. In PS *vs.* non-PS groups, PCI > 20 was 75.0% *vs.* 59.3% (*P* = 0.049); CC 2–3 was 63.3% *vs.* 32.6% (*P* < 0.001); ascites > 1000 mL was 60.0% *vs.* 31.4% (*P* = 0.003). There were no significant differences in other indicators between the two groups (Table 3).

**Table 3:** Major CRS + HIPEC characteristics of MPM patients between PS and non-PS groups

Variables	Non-PS group (*n* = 86)	PS group (*n* = 60)	*P* value
PCI, n (%)			**0.049**
≤ 20	35 (40.7)	15 (25.0)	
> 20	51 (59.3)	45 (75.0)	
CC, n (%)			** < 0.001**
0–1	58 (67.4)	22 (36.7)	
2–3	28 (32.6)	38 (63.3)	
RBC transfusion (U), n (%)			0.167
< 5	70 (81.4)	43 (71.7)	
≥ 5	16 (18.6)	17 (28.3)	
Resected organs, n (%)			0.825
≤ 2	56 (65.1)	38 (63.3)	
> 2	30 (34.9)	22 (36.7)	
Resected peritoneal areas, n (%)			0.704
≤ 5	40 (46.5)	26 (43.3)	
> 5	46 (53.5)	34 (56.7)	
Ascites (mL), n (%)			**0.003**
0	25 (29.1)	10 (16.7)	
0–1000	34 (39.5)	14 (23.3)	
> 1000	27 (31.4)	36 (60.0)	

*CRS* cytoreductive surgery, *HIPEC* hyperthermic intraperitoneal chemotherapy, *MPM* malignant peritoneal mesothelioma, *PS* paraneoplastic syndrome, *PCI* peritoneal cancer index, *CC* completeness of cytoreduction, *RBC* red blood cells

### Analyses on PS-related factors

Univariate analysis revealed the following 8 factors with statistically significant differences between the two groups (*P* < 0.05): PSS, targeted therapy history, KPS, preoperative CA 125 level, vascular tumor embolus, PCI, CC score and ascites. The above factors were included in binary Logistic regression analysis, which identified 3 factors independently associated with PS: preoperative CA 125 level, vascular tumor embolus and CC score (Table 4).

**Table 4:** Multivariate analysis of MPM patients between PS and non-PS groups

Variables	Wald	OR	95% CI	*P* value
Vascular tumor emboli (Yes *vs.* No)	5.709	2.791	1.203–6.477	0.017
CC (2–3 *vs.* 0–1)	10.365	3.287	1.593–6.782	0.001
Preoperative CA 125 level (Increased *vs.* Normal)	6.284	2.921	1.263–6.755	0.012

*MPM* malignant peritoneal mesothelioma, *PS* paraneoplastic syndrome, *OR* odds ratio, *CI* confidence interval

### Perioperative chemotherapy

The timing and type of chemotherapy for MPM patients between the two groups were analyzed. Univariate analysis revealed statistically significant difference in postoperative intravenous (*P* = 0.003) and intraperitoneal (*P* = 0.010) chemotherapy between two groups, while there was no statistically significant difference in preoperative intravenous (*P* = 0.342) and intraperitoneal chemotherapy (*P* = 0.606) (Table 5). According to the number of chemotherapy regimens received by two groups separately (Table 6), we found that pemetrexed combined with platinum was the most used in both preoperative and postoperative intravenous chemotherapy. The most common preoperative intraperitoneal chemotherapy regimen in both groups was platinum monotherapy. There were slight differences between two groups in postoperative intraperitoneal chemotherapy, pemetrexed plus platinum was most common in the non-PS group, while cisplatin was most common in the PS group.

**Table 5:** Timing of chemotherapy of MPM patients between PS and non-PS groups

Variables	Non-PS group (*n* = 86)	PS group (*n* = 60)	*P* value
Preoperative intravenous chemotherapy, n (%)			0.342
No	47 (54.7)	28 (46.7)	
Yes	39 (45.3)	32 (53.3)	
Preoperative intraperitoneal chemotherapy, n (%)			0.606
No	73 (84.9)	49 (81.7)	
Yes	13 (15.1)	11 (18.3)	
Postoperative intravenous chemotherapy, n (%)			**0.003**
No	1 (1.2)	1 (1.7)	
Yes	72 (83.7)	33 (55.0)	
NA	11 (12.8)	18 (30.0)	
Death	2 (2.3)	8 (13.3)	
Postoperative intraperitoneal chemotherapy, n (%)			**0.010**
No	26 (30.2)	13 (21.7)	
Yes	47 (54.7)	21 (35.0)	
NA	11 (12.8)	18 (30.0)	
Death	2 (2.3)	8 (13.3)	

*MPM* malignant peritoneal mesothelioma, *PS* paraneoplastic syndrome

**Table 6:** Types of chemotherapy of MPM patients between PS and non-PS groups^a^

Variables	Non-PS group (*n* = 86)		PS group (*n* = 60)	
Preoperative intravenous chemotherapy, n (%)				
PEM + DDP/CBP/NDP/LBP		35 (40.7)	PEM + DDP/CBP/NDP/OXA/LBP	24 (40.0)
PEM		4 (4.7)	PTX + DDP/CBP	5 (8.3)
PTX + DDP		2 (2.3)	GEM + DDP/CBP	3 (5.0)
Preoperative intraperitoneal chemotherapy, n (%)				
DDP/CBP		6 (7.0)	DDP/NDP/LBP	9 (15.0)
DDP + 5-FU		3 (3.5)	PEM + DDP	1 (1.7)
DTX + DDP/CBP		2 (2.3)		
Postoperative intravenous chemotherapy, n (%)				
PEM + DDP/CBP		49 (57.0)	PEM + DDP/CBP	26 (43.3)
GEM + DDP/CBP/OXA		19 (22.1)	GEM + DDP/CBP/OXA	5 (8.3)
PTX + DDP		6 (7.0)	PEM	4 (6.7)
Postoperative intraperitoneal chemotherapy, n (%)				
PEM + DDP/CBP		23 (26.7)	DDP	12 (20.0)
DDP/CBP/OXA		19 (22.1)	PEM + DDP/CBP	8 (13.3)
DTX		5 (5.8)		

*MPM* malignant peritoneal mesothelioma, *PS* paraneoplastic syndrome, *PEM* pemetrexed, *DDP* cisplatin, *CBP* carboplatin, *NDP* nedaplatin, *OXA* oxaliplatin, *LBP* lobaplatin, *PTX* paclitaxel, *5-FU* fluorouracil, *DTX* docetaxel, *GEM* gemcitabine

^a^One patient may receive multiple chemotherapy regimen

### Survival analysis

**Fig. 1:**
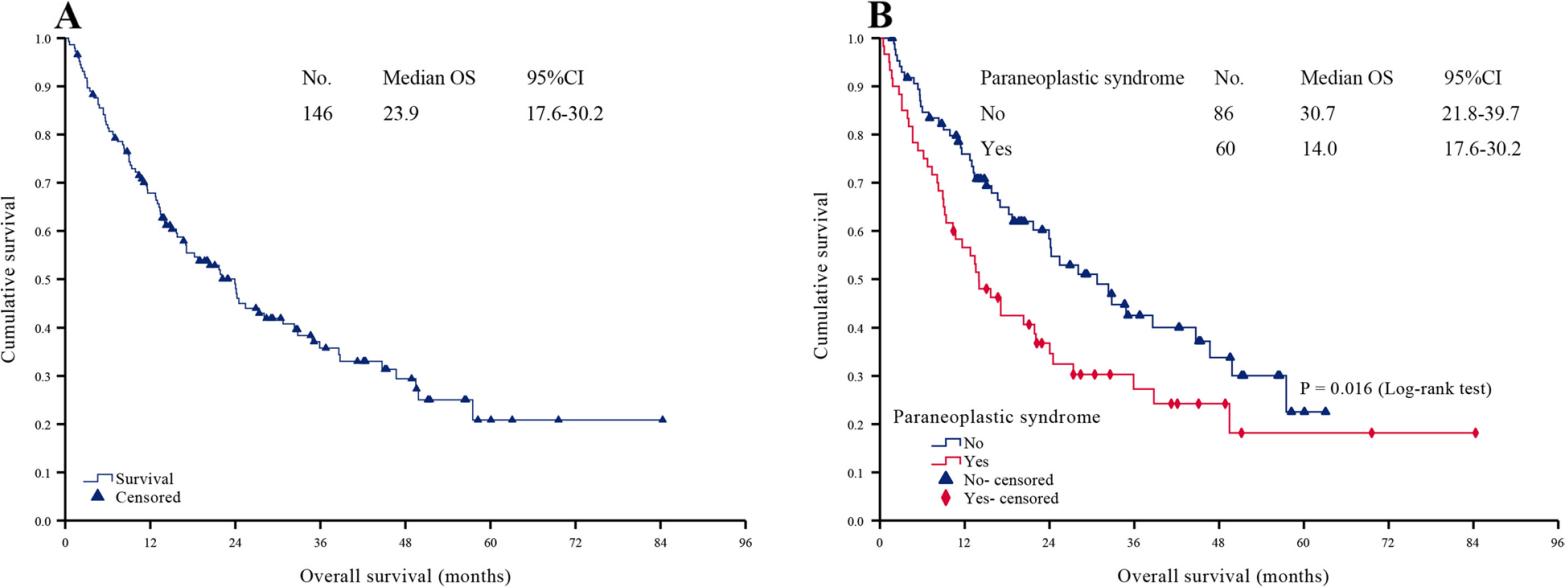
Survival analysis. **A**, Overall survival analysis of all patients. **B**, Survival curve analysis of non-PS group and PS group

At the median follow-up of 36.8 months (95% CI: 27.1–46.4 months), the median OS was 23.9 months (95% CI: 17.6–30.2 months) (Fig. 1A), with 88 patients (60.3%) died, and 58 (39.7%) living. Univariate survival analysis revealed the following 13 clinicopathological factors related to MPM prognosis: surgery history, radiotherapy history, KPS, preoperative CA 125 level, PS (Fig. 1B), PCI, CC score, RBC transfusion, ascites, pathological type, lymphatic metastasis, Ki-67 index and SAEs (all *P* < 0.05).

The above factors were incorporated into the Cox regression model for multivariate analysis, delineating the following 6 independent prognostic factors: KPS, preoperative CA 125 level, PCI, RBC transfusion, Ki-67 index and SAEs (Table 7). PS was not an independent prognostic factor in MPM.

**Table 7:** Multivariate analysis of survival in 146 MPM patients

Variables	Wald	HR	95% CI	*P* value
KPS (≥ 80 *vs.* < 80)	6.252	0.461	0.251–0.846	0.012
PCI (> 20 *vs*. ≤ 20)	11.085	2.576	1.476–4.496	0.001
RBC transfusion (U) (≥ 5 *vs.* < 5)	6.352	1.925	1.157–3.203	0.012
Ki-67 index (> 9% *vs.* ≤ 9%)	8.225	3.857	1.533–9.704	0.004
Preoperative CA 125 level (Increased *vs.* Normal)	8.310	2.331	1.316–4.128	0.004
SAEs (Yes *vs.* No)	11.532	2.116	1.373–3.261	0.001

*MPM* malignant peritoneal mesothelioma, *HR* hazard rate, *KPS* Karnofsky performance status score, *PCI* peritoneal cancer index, *RBC* red blood cell, *HR* hazard rate, *SAEs* serious adverse events

### One typical case presentation of MPM related PS

In March 2022, a 27-year-old male patient was diagnosed as MPM, with a disease history of abdominal distension and fever for 5 months. The abdominal and pelvic computed tomography (CT) examination showed peritoneal thickening and abdominal and pelvic effusion. Peritoneal biopsy showed that mesothelioma could not be excluded.

**Fig. 2:**
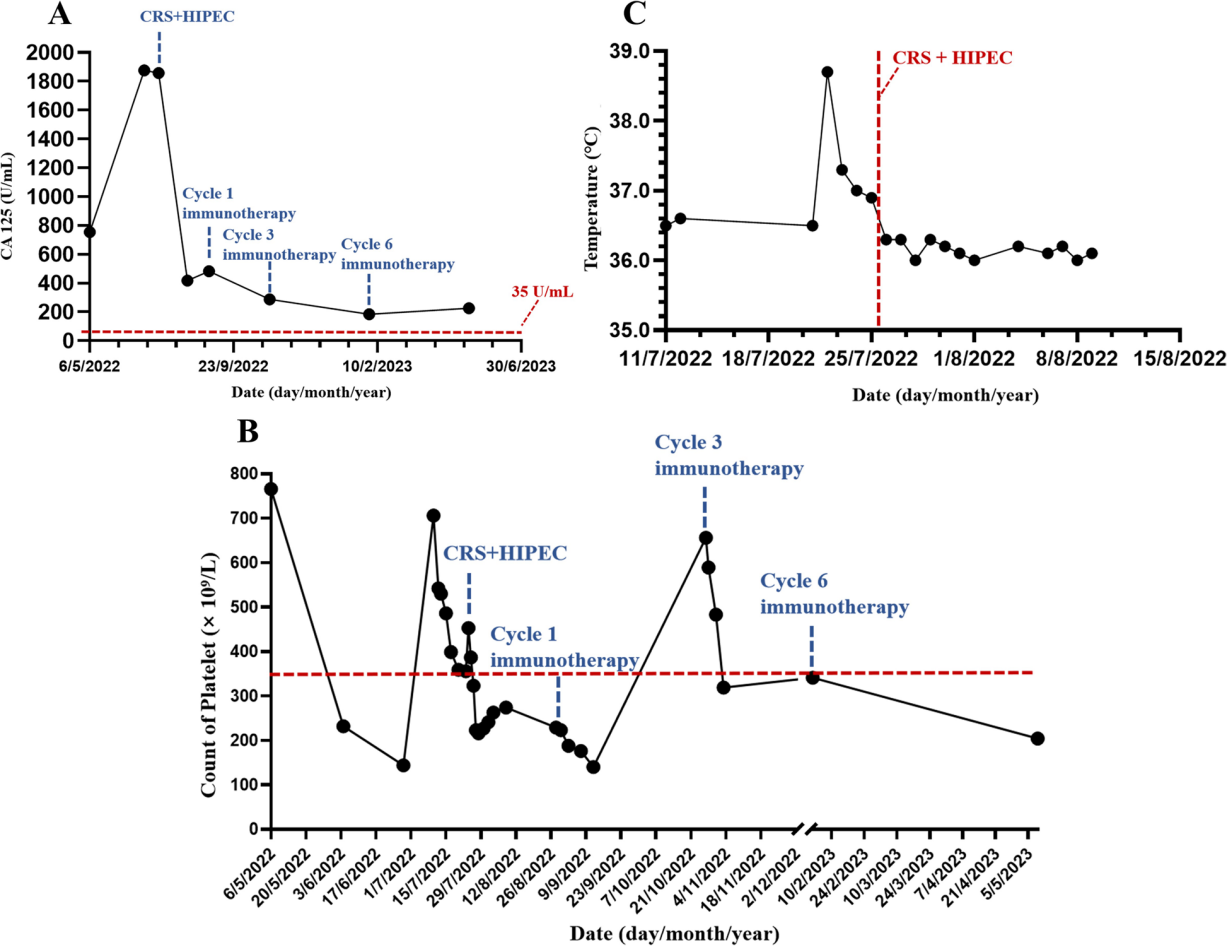
Curves of treatment indicators. **A**, Temperature. **B**, Count of platelet. **C**, CA 125

After admission, the patient continued to have low-grade fever, fatigue, and general discomfort, with the highest temperature of 38.7℃. The related laboratory tests, such as blood and urine routine and culture, C-reactive protein, procalcitonin, ascites culture, and chest X-ray, showed no evidence of infection. After physical cooling, the patient could improve, so neoplastic fever was considered. On July 25, 2022, the patient received CRS + HIPEC and was diagnosed as epithelioid type of MPM. The patient's body temperature was maintained at 36–37 ℃ after surgery, and no fever recurred (Fig. 2A).

In addition, the platelet count of this patient was 766 × 10^9^/L (Upper limit of normal: 350 × 10^9^/L) when he first visited our hospital. The change curves of platelet and CA 125 were analyzed retrospectively (Fig. 2B, C). CA 125 and platelet decreased after each anti-tumor treatment. CA 125 can help to judge the ascites formation and the degree of peritoneal cancer tumor burden, so the decrease of CA 125 indicates the decrease of tumor burden. The change trend of platelet was parallel to the fluctuation of CA 125, suggesting that thrombocytosis was closely related to tumor.

**Fig. 3:**
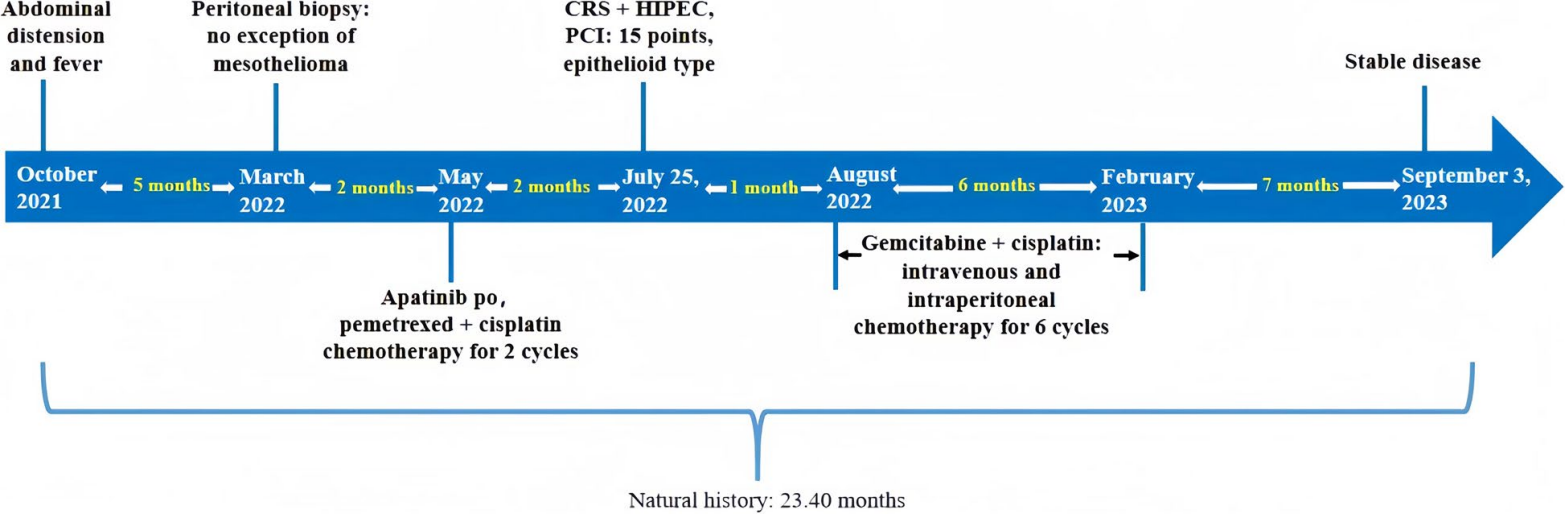
Patient treatment flowchart

By September 3, 2023, the OS of this patient after surgery was 13.47 months (Fig. 3). In conclusion, the case suggests that CRS + HIPEC is the key to treating the primary disease and reduce the tumor burden of MPM with PS. The effect of only symptomatic and supportive treatment for these patients is limited, and CRS + HIPEC as the core anti-tumor treatment should be performed in time.

## Discussion

Among the 146 MPM patients included in this study, 41.1% of the patients developed PS during the disease course, with thrombocytosis (33.6%) and neoplastic fever (9.6%) being the most common. Three factors independently associated with PS were preoperative CA 125 level, vascular tumor embolus, and CC score. The median OS was 23.9 months. Multivariate survival analysis revealed that KPS, preoperative CA 125 level, PCI, RBC transfusion, Ki-67 index, and SAEs were independent prognostic factors for MPM patients. PS was not an independent prognostic factor in MPM.

PS is a heterogenous group of phenomena caused by malignancies influencing the endocrine and immune systems, metabolism, and other mechanisms, not all of which have been identified. According to estimations, PS does not correlate with the stage of tumor or its prognosis [11]. In the past, MPM with PS was considered rare and was often reported as an atypical manifestation of the primary tumor. Specific manifestations can involve multiple organ systems: (1) Hematologic system: thrombocytosis [12], autoimmune hemolytic anemia [13], malignant tumor-associated thrombosis [14], leukemoid reaction [15], recurrent thrombotic thrombocytopenic purpura like syndrome [16]; (2) Endocrine system: hypoglycemia [17] and ectopic corticotrophin secretion syndrome [18]; (3) Urinary system: nephrotic syndrome [19]; (4) Nervous system: myasthenia gravis [20]; (5) Rheumatic system: antiphospholipid syndrome [21] and polymusdoid rheumatism syndrome [22]; (6) Cutaneous system: dermatomyositis [23]; (7) Others: neoplastic fever [24] and cachexia.

This study found that PS in MPM was not rare, the incidence was 41.1%, and the most common was thrombocytosis (33.6%), although it was lower than the previous reports (83%) [25]. The possible mechanism of MPM-related thrombocytosis is that mesothelioma cells persistently secrete interleukin-6, which stimulate thrombopoietin to induce thrombocytosis [25]. Alhamadh et al. [12] pointed out that thrombocytosis is a surrogate marker for tumor aggressiveness and has been associated with poor survival. The second most common PS was neoplastic fever (9.6%). Hermann et al. [9] pointed out that almost any other cancer can cause neoplastic fever, which may be caused by a variety of pyrogen in the body, such as tumor necrosis, interleukin-2 secreted by activated macrophages, and prostaglandins synthesized by tumors.

The risk of PS in MPM patients with vascular tumor embolus was 2.791 times higher than that in patients without vascular tumor embolus. Han et al. [26] found that vascular tumor embolus is an important marker of tumor progression and is often an independent risk factor for the prognosis of malignant tumors. In addition, the risk of PS in MPM patients with increased preoperative CA 125 is 2.921 times higher than that in patients with normal preoperative CA 125. The level of CA 125 is parallel to the growth and decline of the tumor [5]. Therefore, the above studies suggest that MPM with PS often indicates that the primary tumor is at the advanced stage and predicts a large tumor burden and poor prognosis. This was consistent with a much higher proportion of PCI > 20 in the PS group (75.0%) than in the non-PS group (59.3%), which also determined that MPM with PS was more likely to have incomplete cytoreduction (CC 2–3: 63.3% in PS group *vs.* 32.6% in non-PS group).

We also analyzed the timing and type of chemotherapy between PS and non-PS groups, and discovered that the rate of postoperative chemotherapy was higher in the non-PS group than in the PS group, which may be related to the higher postoperative mortality in the PS group. We also found that pemetrexed combined with platinum was the most used regimen during the perioperative period of MPM patients, as consensus suggests.

At present, there is no consensus on the prognostic impacts of PS in cancer patients. Bilynsky et al. [11] pointed out that PS could not predict the treatment outcome of the underlying malignancy. However, Agarwala [10] pointed out that the severity of the syndrome may parallel the activity of the associated tumor and in some instances can be used to follow the clinical course of the disease. In this study, univariate analysis showed that the median OS in non-PS group was 30.7 months, which was significantly longer than that in PS group (14.0 months) (*P* = 0.016). Cox regression results showed that KPS, preoperative CA 125 level, PCI, RBC transfusion, Ki-67 index, and SAEs were independent prognostic factors for MPM patients, which was similar to previous studies [27]. However, PS was not included in the above independent prognostic factors, which may indicate that timely and standardized treatment may reduce the adverse effects of PS on MPM patients. Therefore, it is expected to enhance the awareness of MPM-related PS, improve early diagnosis and treatment, could help further improve the survival of patients, and turn the current adverse prognostic factors into "favorable factors".

There are some limitations in this study. Clinicians have inadequate understanding of PS, incomplete history collection, incomplete examination, and one-sided analysis confined to specialist diagnosis, which leads to the neglect of many abnormal symptoms. In addition, this was a single-center study, the sample size is limited, and the relevant results could not be thoroughly investigated. It is necessary to expand the sample size and include multi-center studies for further verification.

## Conclusion

PS in MPM is not rare, and often indicates that the primary tumor is at the advanced stage. MPM patients with PS have a large tumor burden and high surgical difficulty, leading to poor prognosis. Therefore, improving the understanding of MPM-related PS, early detection, early diagnosis, and early treatment are the key to improving the prognosis of patients.

## Data Availability

The datasets used and/or analyzed during the current study are available from the corresponding author on reasonable request.

## References

[1] Sun L, Li C, Gao S (2022). Diffuse malignant peritoneal mesothelioma: a review. Front Surg.

[2] Broeckx G, Pauwels P (2018). Malignant peritoneal mesothelioma: a review. Transl Lung Cancer Res.

[3] Kusamura S, Baratti D, De Simone M, et al. Diagnostic and therapeutic pathway in diffuse malignant peritoneal mesothelioma. Cancers (Basel). 2023;15(3):662. 10.3390/cancers15030662 PMC9913096 36765620

[4] Chun CP, Song LX, Zhang HP (2023). Malignant peritoneal mesothelioma. Am J Med Sci.

[5] Malpica A (2023). Peritoneal mesothelioma-an update. Adv Anat Pathol.

[6] Schütte K, Trautmann-Grill K (2022). Diagnostics and treatment of clinically relevant paraneoplastic syndromes. Schmerz.

[7] Peritoneal Tumor Committee of Chinese anti-Cancer Association, Tumor Hyperthermia Committee of Chinese anti-Cancer Association, Tumor Hyperthermia Committee of Beijing Cancer Prevention and Control Society (2021). Chinese expert consensus on diagnosis and treatment of diffuse malignant peritoneal mesothelioma. Natl Med J China.

[8] Pelosof LC, Gerber DE (2010). Paraneoplastic syndromes: an approach to diagnosis and treatment. Mayo Clin Proc.

[9] Zell JA, Chang JC (2005). Neoplastic fever: a neglected paraneoplastic syndrome. Support Care Cancer.

[10] Agarwala SS (1996). Paraneoplastic syndromes. Med Clin North Am.

[11] Bilynsky BT, Dzhus MB, Litvinyak RI (2015). The conceptual and clinical problems of paraneoplastic syndrome in oncology and internal medicine. Exp Oncol.

[12] Alhamadh MS, Alanazi RB, Wadaan OM (2023). Thrombocytosis as a paraneoplastic syndrome in metastatic malignant peritoneal mesothelioma of biphasic morphology mimicking ovarian adenocarcinoma: a case report. Clin Case Rep.

[13] Selleslag DL, Geraghty RJ, Ganesan TS (1989). Autoimmune haemolytic anaemia associated with malignant peritoneal mesothelioma. Acta Clin Belg.

[14] Banayan S, Hot A, Janier M (2006). Malignant mesothelioma of the peritoneum as the cause of a paraneoplastic syndrome: detection by 18F-FDG PET. Eur J Nucl Med Mol Imaging.

[15] Thakral B, Loghavi S (2020). Marked paraneoplastic leukemoid reaction in a patient with mesothelioma mimicking a myeloid neoplasm. Blood.

[16] Socola F, Loaiza-Bonilla A, Bustinza-Linares E (2012). Recurrent thrombotic thrombocytopenic purpura-like syndrome as a paraneoplastic phenomenon in malignant peritoneal mesothelioma: a case report and review of the literature. Case Rep Oncol Med.

[17] Tozawa K, Tamai H, Inoue K (2011). Case report; malignant peritoneal mesothelioma with hypoglycemia suggestive of paraneoplastic syndrome. Nippon Naika Gakkai Zasshi.

[18] Mendoza CF, Ontiveros P, Xibillé DX (2015). Ectopic ACTH secretion (EAS) associated to a well-differentiated peritoneal mesothelioma: case report. BMC Endocr Disord.

[19] Dogan M, Ozal G, Savas B (2012). Malign peritoneal mesothelioma with nephrotic syndrome. Bratisl Lek Listy.

[20] Malpica A, Euscher ED, Marques-Piubelli ML (2021). Malignant mesothelioma of the peritoneum in women: a clinicopathologic study of 164 cases. Am J Surg Pathol.

[21] Ozkan M, Eser B, Er O (2004). Antiphospholipid syndrome associated with malignant mesothelioma presenting with superior vena cava thrombosis: a case report. Clin Appl Thromb Hemost.

[22] Ide Y, Yuki T, Taooka Y (2020). Malignant peritoneal mesothelioma presenting with polymyalgia rheumatica-like syndrome. Intern Med.

[23] Von Hirschhausen R, Clemens M (1990). Paraneoplastic dermatomyositis in peritoneal mesothelioma. Med Klin (Munich).

[24] Hermann J, Bajko G, Stajgis M (2012). Fever of unknown origin: a clinical mask of malignant peritoneal mesothelioma. Contemp Oncol (Pozn).

[25] Kimura N, Ogasawara T, Asonuma S (2005). Granulocyte-colony stimulating factor- and interleukin 6-producing diffuse deciduoid peritoneal mesothelioma. Mod Pathol.

[26] Han HM, Wang P, Zhu B (2021). Clinical study of vascular invasion in stage II colorectal cancer. J Prac Oncol.

[27] Su YD, Zhao X, Ma R (2023). Establishment of a Bayesian network model to predict the survival of malignant peritoneal mesothelioma patients after cytoreductive surgery plus hyperthermic intraperitoneal chemotherapy. Int J Hyperthermia.

